# From Parallel Programming to Bidirectional Crosstalk: The Brain–Kidney Axis in Cardiovascular–Kidney–Metabolic Syndrome

**DOI:** 10.3390/antiox15060769

**Published:** 2026-06-19

**Authors:** Chien-Ning Hsu, You-Lin Tain

**Affiliations:** 1 Department of Pharmacy, Kaohsiung Municipal Ta-Tung Hospital, Kaohsiung 801, Taiwan; cnhsu@cgmh.org.tw; 2 Department of Pharmacy, Kaohsiung Chang Gung Memorial Hospital, Kaohsiung 833, Taiwan; 3 School of Pharmacy, Kaohsiung Medical University, Kaohsiung 807, Taiwan; 4 Department of Pediatrics, Kaohsiung Chang Gung Memorial Hospital, Kaohsiung 833, Taiwan; 5 College of Medicine, Chang Gung University, Taoyuan 333, Taiwan; 6 Doctoral Program of Clinical and Experimental Medicine, National Sun Yat-Sen University, Kaohsiung 804, Taiwan

**Keywords:** brain–kidney axis, cardiovascular–kidney–metabolic syndrome, chronic kidney disease, cardiovascular disease, Developmental Origins of Health and Disease (DOHaD), gut microbiota, oxidative stress

## Abstract

Cardiovascular–kidney–metabolic (CKM) syndrome is a systemic, interdependent disorder arising from the convergence of metabolic dysfunction, chronic kidney disease, and cardiovascular pathology. Anchored in the Developmental Origins of Health and Disease (DOHaD) framework, this review advances a “parallel hit” model, primarily based on evidence from experimental animal studies, particularly rodent models, posited that early-life environmental insults concurrently program structural and functional vulnerabilities in both renal and central nervous system hubs. These early perturbations prime susceptibility long before clinical manifestations emerge. CKM progression is conceptualized as a two-stage trajectory, with an initial phase of parallel programming affecting kidney and brain development, followed by a transition to maladaptive bidirectional crosstalk. In the later phase, heightened efferent sympathetic outflow and aberrant afferent renal signaling—potentiated by uremic toxin accumulation, neuroinflammation, and blood–brain barrier disruption—drive a self-perpetuating cycle that accelerates cardiorenal and metabolic injury. Key integrative mechanisms, including oxidative stress, chronic low-grade inflammation, mitochondrial dysfunction, and gut microbiota dysbiosis, serve as convergent pathways linking early-life exposures to adult CKM phenotypes. These pathways not only sustain disease progression but also represent actionable therapeutic targets. Importantly, this framework underscores the translational potential of early-life “reprogramming” strategies. Interventions such as precision nutrition, antioxidant supplementation, microbiota-directed therapies (including prebiotics, probiotics, and postbiotics), and mechanism-based pharmacotherapies may mitigate or reverse maladaptive programming. However, much of the current mechanistic evidence remains preclinical, and further human studies are needed to validate these pathways and therapeutic approaches. Collectively, this dual-hub paradigm reframes CKM syndrome as a life-course continuum rather than a late-stage comorbidity cluster, emphasizing the necessity of early, mechanism-driven interventions to stabilize the brain–kidney axis and improve long-term cardiovascular–kidney–metabolic outcomes.

## 1. Introduction

Cardiovascular disease (CVD) remains the leading cause of mortality worldwide, yet its management is increasingly complicated by the emergence of cardiovascular–kidney–metabolic (CKM) syndrome [1]. Historically, CVD, chronic kidney disease (CKD), and diabetes were considered distinct entities; however, the American Heart Association now defines CKM syndrome as a systemic disorder characterized by complex, interrelated interactions among metabolic risk factors, CKD, and the cardiovascular system [2]. Within this network, pathophysiological processes propagate across organs, generating cardiovascular risk that exceeds the sum of individual conditions [3]. This paradigm shift towards multidimensional frameworks identifies dynamic, bidirectional interdependence between different organ systems and highlights oxidative stress as an important integrative mechanism linking environmental exposures to multi-organ dysfunction.

This system perspective aligns with the Developmental Origins of Health and Disease (DOHaD) hypothesis, which posits that early-life environmental exposures program persistent, multi-organ vulnerability, predisposing individuals to noncommunicable diseases across the life course [4,5]. Maternal insults—including nutritional imbalance, environmental chemicals, maternal illness, and inflammation [6,7,8,9]—act as redox-disrupting stimuli within a One Health context, reflecting the interface between environmental change and human disease. These exposures disrupt organogenesis through mechanisms such as oxidative stress [10], aberrant renin–angiotensin system (RAS) activation [11], sympathetic overactivity [12], reduced nephron endowment [13], glucocorticoid programming [14], and gut microbiota dysbiosis [15]. Among these, oxidative stress represents a primary upstream driver, mediating mitochondrial dysfunction, impaired antioxidant defenses, and redox-sensitive epigenetic modifications. These processes induce parallel programming effects on both the kidney and brain, establishing a latent state of vulnerability with persistently altered neurohumoral setpoints [16]. This “parallel hit” model reflects a shared, exposure-driven initiation phase in which dual-organ vulnerability is established without requiring inter-organ signaling [17].

A central advance in systems biology is the recognition that the brain–kidney axis functions as a tightly coupled, bidirectional network [18,19,20]. Brain-to-kidney signaling, mediated by central autonomic circuits, regulates sympathetic outflow, promoting renal vasoconstriction, sodium retention, and RAS activation. Reciprocally, kidney-to-brain signaling—via afferent neural inputs and circulating uremic toxins—modulates central pathways, amplifying sympathetic activity and inducing neuroinflammation [21]. Evidence from experimental and translational studies suggests that these reciprocal interactions may contribute to the transition from parallel organ injury to bidirectional cross-talk, potentially establishing a self-reinforcing cycle that promotes multiorgan dysfunction over time.

**Figure 1:**
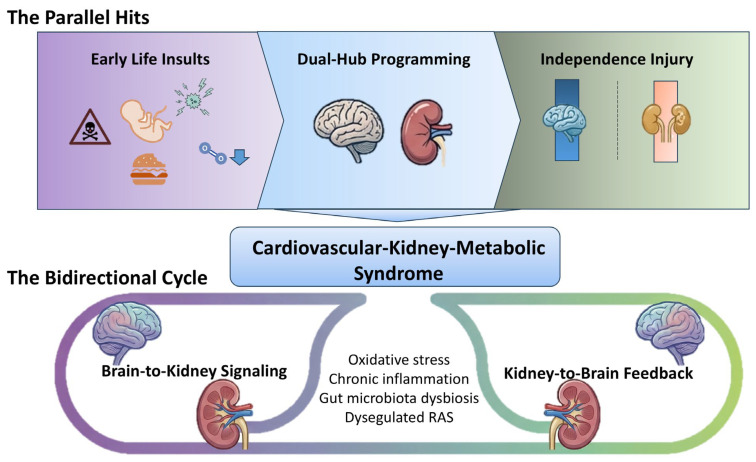
Developmental programming and proposed transition to bidirectional brain–kidney crosstalk in CKM syndrome. Schematic of a hypothesized two-stage trajectory of cardiovascular–kidney–metabolic (CKM) syndrome. Early-life environmental insults are proposed to induce parallel programming in the kidney and brain, creating latent vulnerability characterized by nephron deficit, neuroendocrine reprogramming, oxidative stress, and epigenetic alterations, initially without direct inter-organ communication. With disease progression, it is hypothesized that disruption of barrier systems, particularly the blood–brain barrier, enables transition to bidirectional crosstalk. Central efferent pathways increase sympathetic outflow, driving renal vasoconstriction, sodium retention, and renin–angiotensin system (RAS) activation, while renal afferent signaling—via uremic toxins and neural inputs—amplifies central inflammation and neurohumoral dysregulation. This reciprocal interaction is hypothesized to establish a feed-forward cycle that promotes CKM syndrome.

Mechanistically, convergent mediators—including oxidative stress and inflammation—have been implicated as shared pathways linking early-life insults to long-term cardiovascular, renal, and metabolic vulnerability. This process may be further influenced by coordinated epigenetic modifications across the brain and kidney, reinforcing a dual programming phenotype. Together, these mechanisms support the hypothesis that CKM syndrome progresses via a two-stage trajectory: an initial phase of parallel developmental programming, followed by dynamic bidirectional crosstalk that could contribute to disease progression (Figure 1).

In this narrative review, we propose a conceptual framework in which the brain and kidney function as co-regulators of systemic homeostasis. Their interactions—established early in life and progressively reinforced—may represent a central integrative axis in CKM pathogenesis, with oxidative stress serving as one of several interconnected molecular mechanisms linking environmental drivers to organ-level dysfunction. Across the life course, early programmed susceptibility interacts with postnatal “second hits,” including obesity, high salt intake, and chronic stress, thereby accelerating the emergence of clinical phenotypes such as hypertension, CKD, and insulin resistance [22,23,24]. Although direct validation in humans remains limited, this life-course framework reframes CKM syndrome as a continuum originating in development rather than a late-stage disorder, highlighting early-life intervention and modulation of brain–kidney crosstalk as promising strategies to mitigate long-term risk.

## 2. Early-Life Programming: Parallel Hits on Two Hubs

The developmental trajectory of CKM syndrome is established during critical embryonic and early postnatal windows, when both the brain and kidney are highly immature and vulnerable to environmental perturbations. Evidence from the Dutch Hunger Winter and the Dutch Famine Birth Cohort Study demonstrates that gestational undernutrition induces a coordinated, multi-organ phenotype in adulthood, including coronary heart disease, obesity, CKD, hypertension, and neuropsychiatric disorders [25,26].

Excess glucocorticoid (GC) exposure represents a central programming mechanism. Clinically, synthetic GCs such as dexamethasone are administered to promote fetal lung maturation [27], yet both pharmacologic exposure and stress-induced maternal GC excess—often exacerbated by reduced placental 11β-hydroxysteroid dehydrogenase type 2 (11β-HSD2) activity—act as potent developmental stressors [14], increasing long-term risks of CKD and neuropsychiatric disease [28,29]. Rather than sequential organ injury, these shared insults impose “parallel hits” on two central regulatory hubs—the metanephric (kidney) and neural (brain) systems—initiating a dual-programming state that underlies CKM syndrome.

### 2.1. Kidney Programming

Kidney programming is characterized by early-life insults during nephrogenesis that induce a permanent nephron deficit and long-lasting structural and functional alterations within the metanephric hub [12,30]. Dysregulated placental signaling in compromised pregnancies—characterized by inflammatory cytokines, hormonal imbalance, and immune dysfunction—disrupts fetal–maternal tolerance and promotes a pro-inflammatory intrauterine milieu [31]. In parallel, maternal conditions such as obesity and gestational diabetes induce chronic low-grade inflammation [32,33], which epigenetically programs fetal gene expression, promotes coordinated multi-organ dysfunction, and increases long-term susceptibility to CKM syndrome [34,35]. The developing kidney is particularly vulnerable to oxidative stress during this critical window [36]. Maternal undernutrition, metabolic disorders, medication exposure, smoking, and environmental toxicants increase reactive oxygen species (ROS), impair ureteric bud branching, and reduce nephron endowment [10]. Given that nephrogenesis is completed by approximately 36 weeks of gestation, these insults have irreversible consequences. Reduced nephron number necessitates compensatory single-nephron hyperfiltration, initiating intrarenal inflammation and fibrosis that predispose to CKD [37]. This adaptive hyperfiltration increases intraglomerular pressure, leading to progressive injury of the renal filtration barrier—comprising endothelial cells, the basement membrane, and podocytes—thereby increasing permeability and manifesting as microalbuminuria, an early indicator of systemic microvascular dysfunction. Persistent protein leakage further amplifies tubular inflammation and tubulointerstitial fibrosis, accelerating functional decline of the programmed kidney.

Mechanistically, the programmed kidney acts as a dynamic regulatory node. In compromised pregnancy, elevated asymmetric dimethylarginine (ADMA) reduces nitric oxide (NO) bioavailability and shifts the fetal redox state [38,39], driving sustained activation of the intrarenal RAS, including upregulation of angiotensinogen, angiotensin converting enzyme (ACE), and angiotensin II type 1 receptor (AT1R) [11]. Epigenetic modifications further stabilize this phenotype, with hypomethylation of RAS-related and sodium transporter genes leading to persistent overexpression of NKCC2 and Na^+^/K^+^-ATPase [40,41]. These changes promote sodium retention, salt sensitivity, and chronic inflammation, establishing a trajectory toward hypertension, CKD, and broader CKM syndrome in later life.

### 2.2. Brain Programming

In parallel, early-life insults induce persistent neuronal (or brain) programming within central autonomic and neuroendocrine circuits [42,43]. Excess GC exposure and adverse intrauterine environments remodel the central autonomic network and HPA axis, resetting neurohumoral setpoints toward chronic sympathetic overactivity [44]. Epigenetic mechanisms play a key role in stabilizing these changes. Hypomethylation of hypothalamic AT1R enhances central angiotensin signaling, amplifying sympathetic drive. In metabolic regulatory circuits, hypermethylation of the POMC (Pro-opiomelanocortin) promoter suppresses anorexigenic signaling, predisposing to obesity and metabolic dysregulation in a rat model [45]. Additionally, epigenetic downregulation of the glucocorticoid receptor (NR3C1) in the hippocampus impairs negative feedback of the HPA axis, resulting in sustained stress hyperreactivity [46]. Human data support these findings, as prenatal famine exposure is associated with persistent DNA methylation signatures that extend into adulthood [47], while circulating microRNAs—such as hypoxia-responsive miR-210 and inflammation-related miR-125a—reflect systemic programming linked to CKM risk [48]. Together, these neuroepigenetic alterations contribute to a sustained state of autonomic imbalance and metabolic vulnerability.

### 2.3. The Blood–Brain Barrier as a Regulatory Gatekeeper

Concurrently, the blood–brain barrier (BBB)—a highly specialized neurovascular interface formed by endothelial tight junctions and supported by astrocytes and pericytes—serves as a critical regulatory gatekeeper within the brain–kidney axis [49]. In early life, shared redox imbalance and inflammatory programming establish latent vulnerability of BBB integrity. Rather than acting as a passive barrier, the BBB functions as a dynamic interface that regulates “kidney-to-brain” signaling and governs the transition from silent parallel programming to active inter-organ communication. Although initial programming occurs in parallel, BBB dysfunction represents a pivotal mechanistic switch that enables convergence of the brain and kidney into a tightly coupled, bidirectional network driving CKM progression. Through the efferent limb, programmed central circuits increase sympathetic outflow, promoting renal vasoconstriction, sodium retention, and inflammation, thereby accelerating kidney injury. Conversely, through the afferent limb, the programmed kidney communicates with the central nervous system (CNS). Disruption of the BBB facilitates the entry of renal-derived inflammatory and metabolic mediators into central autonomic nuclei, while renal ischemia and structural injury engage afferent signaling pathways to the brainstem (nucleus tractus solitarius, NTS) and hypothalamus (paraventricular nucleus, PVN), thereby resetting blood pressure (BP) regulation and further amplifying sympathetic outflow. This reciprocal interaction establishes a feed-forward vicious cycle in which neural and renal dysfunction reinforce one another, driving the transition from latent developmental programming to overt CKM phenotypes, including hypertension, CKD, insulin resistance, and CVD.

## 3. Reciprocal Brain–Kidney Crosstalk in CKM Syndrome

**Figure 2:**
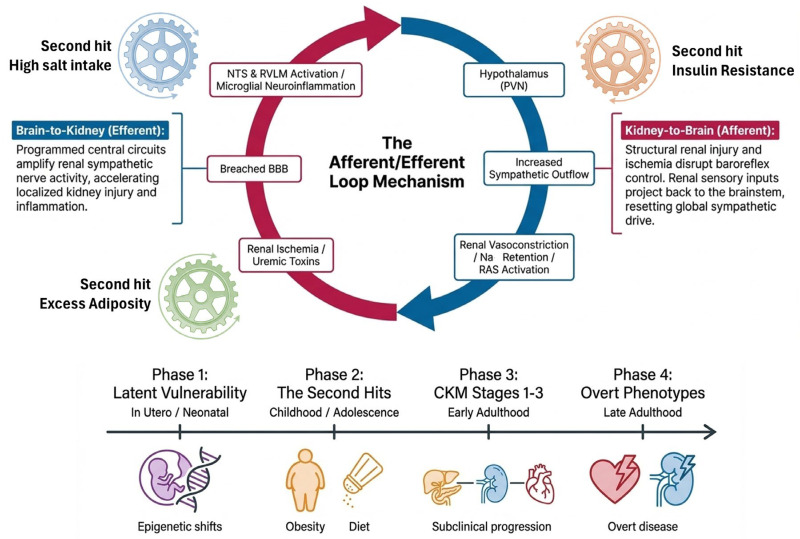
Schematic of the brain–kidney axis in cardiovascular–kidney–metabolic (CKM) syndrome, illustrating a bidirectional afferent–efferent loop. Afferent signals from renal injury, ischemia, and uremic toxins impair baroreflexes and activate brainstem nuclei (NTS, RVLM), triggering hypothalamic (PVN)-mediated sympathetic activation and neuroinflammation. Efferent output increases sympathetic drive, causing renal vasoconstriction, sodium retention, RAS activation, inflammation, and BBB disruption. Second hits, including obesity, high salt intake, and insulin resistance, further amplify neurohumoral dysregulation. The timeline depicts CKM progression from early-life epigenetic programming (Phase 1) through environmental exposure (Phase 2), subclinical injury (Phase 3), to overt disease (Phase 4), reflecting maladaptive brain–kidney crosstalk.

CKM syndrome is categorized into Stages 1–4, reflecting increasing severity across its spectrum: Stage 1, excess or dysfunctional adiposity; Stage 2, established metabolic and renal risk factors; Stage 3, subclinical cardiovascular injury or high-risk states; and Stage 4, overt CVD with or without kidney failure. Mechanistically, Figure 2 illustrates the bidirectional brain–kidney axis as a central driver of disease progression. In the afferent (kidney-to-brain) pathway, renal injury, ischemia, and uremic toxins impair baroreflexes and activate brainstem nuclei (NTS, RVLM), inducing neuroinflammation and hypothalamic (PVN)–mediated sympathetic activation. In the efferent (brain-to-kidney) pathway, increased sympathetic outflow promotes renal vasoconstriction, sodium retention, RAS activation, inflammation, and BBB disruption. “Second hits,” including obesity, high salt intake, and insulin resistance, further amplify these neurohumoral disturbances.

At the systemic level, interconnected pathways—oxidative stress, chronic inflammation, gut microbiota dysbiosis, and dysregulated RAS [18,19,20]—translate early-life exposures into long-term, multi-organ vulnerability. Figure 2 further conceptualizes CKM progression from early-life epigenetic programming (Phase 1), through environmental and dietary exposures (Phase 2), to subclinical organ injury (Phase 3), and finally overt disease (Phase 4), underscoring the role of maladaptive brain–kidney crosstalk in driving CKM phenotypes.

### 3.1. Obesity: Brain–Kidney Axis Amplification

Obesity—particularly excess or dysfunctional adiposity—is a central initiating and amplifying factor in CKM syndrome, corresponding to Stage 1 of its progression. Early-life neuronal and renal programming may increase susceptibility to obesity later in life. At the neural level, maternal overnutrition induces epigenetic modifications in hypothalamic circuits (e.g., POMC), disrupting appetite regulation and increasing sympathetic outflow [45]. In parallel, kidney programming—characterized by heightened inflammatory signaling and intrarenal RAS activation—reinforces metabolic and hemodynamic dysregulation, collectively promoting an obesogenic phenotype [50,51]. In obesity, reciprocal brain–kidney communication involves both efferent and afferent pathways. The efferent brain-to-kidney axis, driven by hypothalamic dysregulation, enhances renal sympathetic nerve activity, increasing sodium reabsorption and activating intrarenal RAS, thereby amplifying metabolic and hemodynamic stress [52]. Notably, adipose expansion includes ectopic fat deposition within and around the kidney [53], where perirenal and intrarenal adipose tissue exerts local paracrine effects that promote inflammation, oxidative stress, and renal hemodynamic impairment. Concurrently, adipose tissue functions as an active endocrine and inflammatory organ, releasing cytokines (e.g., TNF-α, IL-6) and activating pathways such as the NLRP3 inflammasome, which drive systemic inflammation, endothelial dysfunction, and insulin resistance [54]. This inflammatory state has been associated with blood–brain barrier (BBB) dysfunction and altered central metabolic regulation. In both human and experimental obesity, elevated ADMA further contributes to endothelial and BBB dysfunction, promoting neuroinflammation [55,56], and is associated with CKM-related phenotypes, including stroke, hyperlipidemia, CVD, and CKD [57]. Collectively, ectopic and systemic adipose tissue act as key inflammatory hubs that propagate CKM progression through integrated neurohormonal and brain–kidney axis signaling.

### 3.2. CKD-Induced Neurotoxicity

CKD within CKM syndrome extends beyond its traditional role in filtration to actively reshape central nervous system function. CKD activates the afferent limb of the brain–kidney axis, where renal sensory inputs and circulating uremic toxins converge on brainstem (NTS, RVLM) and hypothalamic nuclei [58]. These signals disrupt baroreflex control and enhance sympathetic and vasopressinergic outflow through mechanisms involving oxidative stress, NO deficiency, and inflammation [59]. This establishes a maladaptive “Kidney-to-Brain” feedback loop that sustains neurohumoral activation and accelerates multi-organ deterioration. A critical mediator in this crosstalk is the dysregulation of microbiota-derived metabolites, characterized by the concurrent accumulation of uremic toxins and the depletion of protective factors [60]. As kidney filtration declines, protein-bound uremic toxins such as indoxyl sulfate (IS), p-cresyl sulfate (pCS), and trimethylamine N-oxide (TMAO) accumulate in the systemic circulation. There is increasing evidence to show that uremic toxins, IS and pCS, have a neurotoxic effect [61]. TMAO, generated via microbial metabolism of dietary choline, accumulates in CKD due to reduced renal clearance and acts as a neurovascular stressor leading to neuroinflammation [62]. While its association with cardiovascular risk is partly confounded by renal function, TMAO promotes inflammation and endothelial dysfunction, contributing to astrocyte and lymphatic endothelial injury within the cerebral lymphatic system [63]. These toxins breach the BBB, which is often already compromised by systemic inflammation and CKD-induced endothelial dysfunction. Mechanistically, tryptophan-derived uremic toxins like IS act via the aryl hydrocarbon receptor (AhR) to activate microglial cells and astrocytes, inducing a state of sustained neuroinflammation [64].

Beyond uremic toxins, CKD-driven neuroinflammation is reinforced by persistent NF-κB/ROS signaling, mitochondrial dysfunction with impaired mitophagy and release of damage-associated molecular patterns, and cellular senescence with a senescence-associated secretory phenotype (SASP), all of which propagate inflammatory signaling to the CNS [65]. These converging mechanisms establish a chronic neuroinflammatory milieu that underlies cognitive decline and central autonomic dysregulation within CKM syndrome.

### 3.3. Gut Integration in the Brain–Kidney Axis

Uremic neurotoxicity within the brain–kidney axis is further amplified by a profound reduction in short-chain fatty acids (SCFAs), key microbial metabolites such as butyrate and propionate [66]. Under physiological conditions, SCFAs preserve BBB integrity by enhancing tight junction proteins and regulating central inflammatory responses via histone deacetylation and G-protein-coupled receptor signaling [66]. In CKD, the loss of SCFA-producing bacteria removes these protective mechanisms, leading to BBB disruption, increased neuroinflammation, and a shift toward a pro-oxidative central environment that facilitates neuronal injury and cognitive decline [67].

Concurrently, gut dysbiosis promotes the accumulation of uremic toxins, further exacerbating endothelial dysfunction and neuroinflammatory activation. These alterations establish a maladaptive feedback loop within the brain–kidney axis: neuroinflammation enhances efferent sympathetic outflow to the kidney, driving renal vasoconstriction, sodium retention, and progressive nephron loss. This in turn worsens renal clearance and further amplifies gut-derived toxin accumulation, reinforcing the cycle of injury. Collectively, these findings underscore that cognitive impairment in CKD reflects a direct consequence of disrupted gut–brain–kidney axis signaling rather than an incidental finding [68].

### 3.4. Bidirectional Hypertension Loop

Hypertension represents a key clinical outcome of a programmed brain–kidney axis [16], where early-life environmental insults establish latent vulnerability in neurohumoral and renal regulatory systems. Epigenetic modifications during critical developmental windows impair nephrogenesis and recalibrate central sympathetic control, forming a primed but subclinical phenotype. This vulnerability is unmasked and amplified by “second hits” in later life, including high-salt intake, obesity, inflammation, and gut dysbiosis [60,69,70,71]. Hypertension is sustained by heightened sympathetic nervous system (SNS) activity within this axis, where central activation from the PVN and RVLM increases renal sympathetic nerve output, promoting renin release, sodium retention, and renal vasoconstriction [72]. These secondary stressors intensify efferent sympathetic drive, promoting renin release, sodium retention, and renal vasoconstriction [72], while exacerbating nephron-deficit-induced hyperfiltration and intrarenal RAS activation. Concurrently, kidney injury enhances afferent signaling to the brain, disrupting the renorenal reflex [73], amplifying global sympathetic outflow, and establishing a self-perpetuating cycle. Together, this bidirectional amplification transforms early programmed susceptibility into overt, sustained hypertension.

### 3.5. Metabolic Second Hits

Insulin resistance is an upstream driver initiating the CKM syndrome trajectory, followed by downstream metabolic derangements such as dyslipidemia and diabetes [2]. Early neuronal programming of the HPA axis results in elevated basal cortisol levels, which drive insulin resistance, visceral fat deposition, and diabetes across the life course [74]. These metabolic shifts are further complicated by hyperlipidemia, as dysregulated lipid metabolism and high glucose levels trigger local RAS activation within the liver, promoting the development of metabolic dysfunction-associated steatotic liver disease (MASLD) characterized by hepatic inflammation and fibrosis [75]. Central to this progression is dysbiosis-associated “leaky gut,” reflecting disruption of the gut–blood barrier that normally restricts microbial products and toxic metabolites, allowing bacterial endotoxins and metabolites such as TMAO to enter the circulation and stimulate immune activation and cytokine production [60]. These pro-inflammatory cytokines breach the BBB and activate central neuroinflammatory pathways, which further aggravate hypothalamic metabolic control and systemic insulin resistance [76]. Reciprocally, these metabolic risks act as a “second hit” that exacerbates early-life programming in both hubs. Chronic hyperglycemia from diabetes induces profound oxidative stress in the kidney, promoting the accumulation of advanced glycation end-products that cause glomerular hypertrophy and accelerate the transition to clinical CKD [77]. Simultaneously, glucose-mediated endothelial injury compromises the cerebral microvasculature, promoting microvascular dysfunction and increasing susceptibility to ischemic stroke [78]. Impaired vascular integrity and clearance mechanisms, together with reduced renal elimination of uremic toxins, further amplify neurovascular injury and stroke risk [79]. From this perspective, metabolic risks—such as diabetes, hyperlipidemia, and MASLD—act as “second hits” rather than isolated comorbidities, amplifying early-life programming and reinforcing the bidirectional pathological crosstalk between the brain and kidney, thereby accelerating the progression of CKM syndrome.

### 3.6. CVD as the Integrative End-Organ Phenotype in CKM Syndrome

Although CVD is considered the terminal stage of CKM syndrome [2], it also functions as a central hub and force multiplier, integrating metabolic, renal, and neurovascular dysfunction into a unified, self-amplifying disease continuum. It is biologically interconnected with CKD through bidirectional pathways, commonly referred to as cardiorenal syndrome [80]. In the life-course paradigm, this connection is unified by the brain–kidney axis through the Strain Vessel Hypothesis [81]. Both the brain and kidney utilize specialized strain vessels—short, high-pressure arterioles (e.g., cerebral perforating arteries and renal afferent arterioles)—that are exposed to marked hemodynamic stress and are therefore highly susceptible to injury. Albuminuria may represent an early marker of damage in these vessels, reflecting shared microvascular vulnerability that links renal dysfunction, salt-sensitive hypertension, and increased risk of stroke and CVD.

In the brain–kidney axis, programmed renal ischemia and uremic neurotoxicity activate renal afferent pathways projecting to brainstem and hypothalamic centers, resetting baroreflex control and enhancing global sympathetic outflow [18]. This kidney-to-brain feedback loop sustains a self-perpetuating cycle in which heightened sympathetic activity promotes hypertension, cardiac hypertrophy, and arrhythmias, while impaired renal clearance of uremic toxins accelerates vascular injury, arterial stiffness, and cerebral small vessel damage [18]. Within CKM syndrome, these convergent CKM perturbations accelerate cerebrovascular injury, including cerebral small vessel disease and stroke. Stroke therefore represents a key neurovascular endpoint of CKM syndrome [82], reflecting cumulative microvascular damage and neurohumoral dysregulation, and marking the clinical culmination of this interconnected, life-course disease continuum.

## 4. Targeted Intervention of the Brain–Kidney Axis

**Figure 3:**
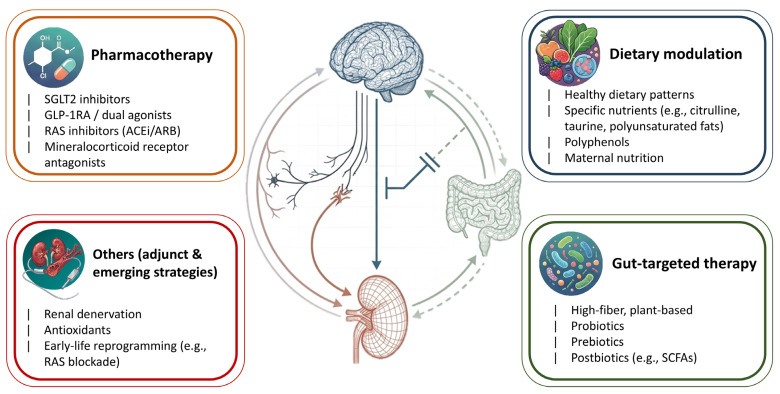
Mechanistic integration and therapeutic targeting of the brain–kidney axis in CKM syndrome. Overview of integrated mechanisms and targeted interventions along the brain–kidney axis. Therapeutic strategies focus on disrupting maladaptive crosstalk through mechanism-based interventions. Pharmacological agents, including SGLT2 inhibitors, GLP-1 receptor agonists, renin–angiotensin system inhibitors, and mineralocorticoid receptor antagonists, attenuate neurohumoral activation and improve redox and inflammatory balance. Dietary modulation, microbiota-targeted therapies, antioxidant strategies, and renal denervation support restoration of axis homeostasis and enable precision modification of CKM progression across the life course.

Therapeutic strategies for CKM syndrome have shifted toward phenotype-guided, mechanism-based pharmacotherapy that targets neurohumoral dysregulation, renal hemodynamic stress, and metabolic signaling as integrated components of the brain–kidney axis [83,84] (Figure 3). Increasingly, these strategies emphasize modulation of neuroinflammation as a central mechanism, whereby CKD-associated inflammatory signaling and uremic toxins converge on the CNS to activate microglia and astrocytes, amplify oxidative stress, and sustain hypothalamic and brainstem-driven sympathetic overactivity. Rather than uniform risk-factor control, this approach leverages agents with multi-organ protective and central–peripheral actions to interrupt maladaptive feedback loops.

### 4.1. SGLT2 Inhibitors

Sodium–glucose cotransporter-2 (SGLT2) inhibitors represent a cornerstone of this paradigm [85]. In large outcome trials such as CREDENCE and DAPA-CKD, they achieved ~30–40% reductions in major renal endpoints while also lowering cardiovascular risk [86,87]. Mechanistically, beyond glucosuria, SGLT2 inhibitors reduce intraglomerular hypertension, attenuate renal afferent signaling, and dampen central sympathetic activation [88]. They also restore systemic redox balance by decreasing mitochondrial and NADPH oxidase-derived ROS [89]. Emerging preclinical evidence suggests potential neuroprotective effects, including attenuation of hippocampal neuroinflammation and modulation of neurodegenerative pathways, although these findings remain insufficiently validated in clinical settings, reinforcing their role in targeting brain–kidney crosstalk [90].

In addition, the clinical perspective of SGLT-based therapy may be broadened by dual SGLT1/2 inhibition (e.g., sotagliflozin) [91]. Beyond renal and cardiovascular effects similar to SGLT2 inhibitors, SGLT1 inhibition in the proximal intestine delays glucose absorption and shifts uptake toward more distal intestinal segments, thereby modifying glycemic delivery to the gut microbiota [92]. This may influence microbial composition and metabolite production, with potential downstream effects on systemic inflammation, metabolic regulation, and gut–heart–kidney–brain axis signaling.

### 4.2. GLP-1 Receptor Agonists

Incretin-based therapies, including GLP-1 receptor agonists (GLP-1RAs) and dual GIP/GLP-1 agonists, act as key metabolic regulators within CKM syndrome, exerting coordinated central and peripheral effects [93]. Preclinical and translational studies suggest that these agents may engage central appetite-regulating circuits via limited blood–brain barrier penetration and gut–brain signaling pathways, particularly involving hypothalamic and brainstem nuclei, thereby improving energy homeostasis. Experimental data also indicate attenuation of microglial activation and NF-κB-associated neuroinflammatory signaling, although these effects remain primarily demonstrated in preclinical models rather than established clinical outcomes, thereby suggesting potential neurobiological relevance along the brain–kidney axis [94]. By directly modulating CNS inflammatory signaling, they help interrupt feed-forward neuroimmune circuits linking metabolic stress to sympathetic activation. Peripherally, incretin therapies promote adipose tissue reduction and reverse obesity-related metabolic dysfunction, including MASLD, representing a disease-modifying approach [95]. They also confer robust cardiovascular protection, improving endothelial function and arterial compliance while significantly reducing major adverse cardiovascular events in large outcome trials. In the kidney, these agents improve glomerular hemodynamics by reducing intraglomerular pressure, while suppressing inflammatory and profibrotic signaling pathways, including MCP-1 and TGF-β1 [96].

### 4.3. RAS Inhibitors

In CKM syndrome, RAS inhibitors provide coordinated neuro–renal protection by targeting both central and peripheral components of the brain–kidney axis [97]. Centrally, AT1R blockers (ARBs) can cross the BBB to suppress microglial activation and neuroinflammation, thereby reducing neurogenic sympathetic outflow and stabilizing central autonomic regulation [98]. Peripherally, RAS blockade attenuates renal inflammation and reduces uremic toxin generation. In parallel, emerging anti-inflammatory strategies—including IL-1/IL-6 axis inhibition and NLRP3 inflammasome targeting—offer additional approaches to suppress upstream cytokine signaling and limit neuroimmune amplification. These approaches may complement established therapies by reducing IL-1β/IL-18–mediated inflammation and pyroptotic signaling. RAS inhibition also acts synergistically with SGLT2 inhibitors and GLP-1RAs to restore redox homeostasis and suppress NF-κB-driven inflammation [99]. In patients with persistent inflammatory–fibrotic CKM phenotypes, finerenone further attenuates profibrotic and proinflammatory signaling, contributing to stabilization of neurohumoral activation [100].

Importantly, long-term RAS blockade may be associated with aldosterone breakthrough or escape [101], characterized by a rebound or persistent increase in circulating aldosterone levels, which may contribute to ongoing target-organ injury despite therapy. In addition, emerging evidence indicates that mineralocorticoid receptor (MR) activation may occur independently of aldosterone, including glucocorticoid-mediated MR activation under conditions of altered 11β-hydroxysteroid dehydrogenase (11β-HSD) type 1 and type 2 activity [102], as well as RAC1-mediated ligand-independent MR activation [103]. These alternative pathways may promote sustained MR signaling in the heart, kidney, brain, and gut, thereby amplifying inflammatory, oxidative, and fibrotic responses [104]. Collectively, these mechanisms further suggest MR activation as a central integrative node in CKM pathophysiology and highlight potential limitations of RAS inhibition alone, underscoring the rationale for combined or downstream MR-targeted therapeutic strategies [105].

### 4.4. Other Pharmacological Approaches

Enhancement of endogenous cytoprotective pathways further strengthens this framework. Nrf2 activators restore redox balance and suppress NF-κB-mediated neuroinflammation, while senolytic therapies reduce senescence-associated secretory phenotype factors that propagate inflammatory signaling to the CNS. In addition, JAK/STAT pathway inhibition provides a central strategy to dampen cytokine-driven signaling across kidney and brain, thereby reducing sympathetic activation and multi-organ inflammation. Notably, no single pharmacotherapy directly restores the BBB, renal filtration barrier, or gut–vascular barrier; however, current agents converge on shared mechanisms—improving endothelial function, reducing oxidative stress, and suppressing inflammation—thereby stabilizing barrier integrity.

Collectively, these therapies converge on attenuation of neuroinflammation, restoration of redox homeostasis, and suppression of chronic sympathetic activation, establishing the brain–kidney axis as a central therapeutic target. By interrupting feed-forward neuroimmune circuits, these strategies offer a unified approach to prevent and treat CKM syndrome. Combination regimens integrating SGLT2 inhibitors, incretin-based therapies, RAS modulation, and emerging immunomodulatory approaches may achieve synergistic disruption of this axis.

### 4.5. Renal Nerve Ablation

Beyond pharmacologic approaches, catheter-based renal nerve ablation (CBRNA) represents a targeted interventional strategy to interrupt the brain–kidney vicious cycle driven by chronic sympathetic overactivity [106]. By targeting the renal nerves residing within or near the adventitial layer of the renal artery, CBRNA aims to attenuate both efferent and afferent renal sympathetic signaling. On the efferent limb (Brain-to-Kidney), this procedure reduces sympathetic-driven renin release from juxtaglomerular cells, decreases tubular sodium reabsorption, and lowers renal vascular resistance, thereby contributing to reductions in chronic volume expansion and blood pressure in selected patient populations [107]. Concurrently, the ablation of the sensory afferent limb (Kidney-to-Brain) is critical, as it prevents pathological signals—often stemming from programmed renal ischemia or inflammation—to central integration hubs such as the PVN and RVLM, as demonstrated primarily in preclinical and physiological studies. This central modulation may contribute to reductions in global sympathetic tone, which has been hypothesized to relate to observed clinical effects extending beyond BP reduction, including metabolic and rhythm-related outcomes; however, these pleiotropic effects remain incompletely established in large-scale clinical studies.

Validated by high-quality randomized controlled trials utilizing radiofrequency, ultrasound, or chemical ablation, CBRNA offers a nondrug-based means to stabilize the CKM continuum by resetting established life-course neurohumoral setpoints. Preclinical evidence from DOCA-salt models underscores that therapeutic success is frequently mediated by the specific interruption of excitatory renal sensory inputs. While CBRNA shows BP-lowering efficacy and emerging evidence in selected cardiovascular and renal populations [108], its impact on long-term multi-organ CKM progression remains incompletely defined, and its optimal integration into the broader CKM framework requires further refinement, potentially through the use of predictive biomarkers—such as urinary cytokines—to identify high-risk subphenotypes most likely to benefit from axis-targeted neuromodulation.

## 5. Early-Life Reprogramming of the Brain–Kidney Axis

Translating the DOHaD framework into clinical practice requires a shift from reactive, organ-specific management to proactive reprogramming of the bidirectional brain–kidney axis. Viewing the brain and kidney as coordinated regulators of early-life programming supports integrated strategies targeting neurohumoral, metabolic, and inflammatory pathways during critical developmental windows (Figure 3). However, much of the current evidence supporting these concepts is derived from experimental and preclinical studies, particularly rodent models, and should be interpreted with appropriate caution regarding human translation.

### 5.1. Maternal Nutrition

Maternal nutrition is a principal determinant of fetal programming and a key modifiable target to prevent offspring CKM syndrome [109]. During gestation and lactation, optimized dietary patterns—exemplified by the Mediterranean diet, characterized by balanced macronutrient composition, high intake of plant-based foods and unsaturated fats, and reduced fructose and saturated fat—are associated with modulation of neurohumoral, metabolic, and inflammatory pathways governing long-term cardiometabolic and renal homeostasis [110,111,112]. Evidence from rodent models, in which nephrogenesis extends into the early postnatal period, demonstrates that early-life nutritional modulation may preserve nephron endowment, is associated with normalization of sympathetic and RAS activity, and may restore coordinated brain–kidney signaling, thereby potentially reducing CKM risk [109].

Mechanistically, dietary reprogramming converges on NO bioavailability, redox balance, epigenetic regulation, and microbiota-derived metabolites. Amino acids such as citrulline and taurine may enhance NO signaling and attenuate oxidative stress [113,114]. Lipid quality is also critical, with polyunsaturated fatty acids being associated with protection against metabolic and vascular dysfunction, whereas saturated fats promote maladaptive programming [115,116]. Micronutrients and methyl donors regulate one-carbon metabolism and DNA methylation [117,118], while antioxidant vitamins and polyphenols (e.g., resveratrol, quercetin) have been shown in preclinical models to suppress oxidative stress, inflammation, and dysbiosis across the brain–kidney axis [119,120,121,122]. Importantly, these interventions are highly context- and timing-dependent; inappropriate strategies, such as caloric restriction, may be associated with maladaptive programming and increase susceptibility to obesity, hypertension, and kidney disease [109]. Thus, effective reprogramming requires precise alignment of nutritional composition, developmental timing, and maternal–fetal context.

### 5.2. Antioxidants

Given the central role of oxidative stress in CKM programming, redox-targeted interventions have emerged as a focused preventive strategy [10]. Restoration of redox homeostasis and NO signaling during early development is critical for nephrogenesis, vascular integrity, and neurohumoral balance [123,124]. Perinatal antioxidant interventions—including NO precursors, selected vitamins, and polyphenols [119,120,125]—have been shown in animal studies to preserve nephron endowment, reduce oxidative injury, and stabilize brain–kidney signaling. Notably, agents such as melatonin, resveratrol, and N-acetylcysteine act beyond direct ROS scavenging by enhancing endogenous antioxidant defenses, modulating NO and hydrogen sulfide pathways, and reprogramming neurohumoral signaling [126,127]. These effects are associated with reduced neuroinflammation, improved mitochondrial function, and stabilization of sympathetic and renal regulation. However, clinical translation remains limited by heterogeneity in timing, dosing, and population characteristics, as well as the risk of disrupting physiological redox signaling with excessive antioxidant exposure [128,129].

### 5.3. Gut-Targeted Interventions

The gut microbiota has emerged as a central regulator of the brain–kidney axis in CKM syndrome [60], providing a critical interface for reprogramming disease trajectories [130]. Diets rich in plant-based and high-fiber foods—including Mediterranean, DASH (Dietary Approaches to Stop Hypertension), and vegetarian patterns—are associated with increased microbial diversity and beneficial metabolite production, thereby potentially improving cardiometabolic and renal outcomes [131,132,133].

Gut-targeted interventions—including probiotics, prebiotics, and postbiotics—may modulate microbial composition and function [134], and could contribute to interruption of intergenerational transmission of CKM risk. Preclinical studies provide strong mechanistic support. Perinatal probiotic supplementation (e.g., *Lactobacillus casei*, *Lactiplantibacillus plantarum*) has been shown in rodent models to enhance microbial diversity and ameliorates hypertension, dyslipidemia, and insulin resistance in offspring [135,136]. Maternal prebiotics such as inulin, oligofructose, and fructooligosaccharides have been reported to protect against hypertension, obesity, fatty liver, and diabetes in rodent models [136,137,138]. Functional foods with prebiotic properties, such as garlic (*Allium sativum*), may confer additional benefits, partly via hydrogen sulfide signaling, enrichment of beneficial taxa (e.g., *Bifidobacterium*, *Lactobacillus*), and increased SCFA production [139]. SCFAs—including acetate, propionate, and butyrate—serve as key postbiotic mediators [140,141], with perinatal supplementation shown in preclinical studies to be associated with prevention or attenuation of programmed hypertension, diabetes, and dyslipidemia in offspring exposed to adverse maternal environments [142,143,144]. Collectively, these findings suggest a causal role for microbiota modulation in CKM prevention and highlight its translational potential during early life.

Pharmacological targeting of the brain–kidney axis has been widely applied in established CKM syndrome, although its role in reprogramming remains less explored. Early-life RAS blockade—using agents such as aliskiren, captopril, or losartan—has been shown in rodent models to reset homeostatic set points and prevent the development of adult hypertension when administered during critical developmental windows (e.g., postnatal weeks 2–4 in rodents) [145,146,147].

## 6. Precision CKM Syndrome: A Brain–Kidney Life-Course Framework

Management of CKM syndrome is evolving from phenotype-led staging models towards precision medicine driven by biological knowledge pertinent to the brain–kidney axis [148,149]. The CKM staging system (Stages 0–4) offers a clinical structure, but disease progression should be viewed more as a continuum of interlinked neuro–renal–metabolic processes. The brain–kidney axis acts as a convergence point in which uremic toxins, inflammatory mediators, and autonomic signaling promote systemic dysfunction. As such, precision strategies thus center around the early identification of maladaptive circuits [150,151], specifically within Stages 0–3 when subclinical organ injury and neurohumoral dysregulation first occur. Precision prediction integrates longitudinal risk modeling with molecular data to capture cumulative disease burden, and the PREVENT equations extend risk estimation to 30 years while incorporating kidney function as a key determinant of cardiovascular and neurological risk [3].

### 6.1. Precision Diagnosis: Molecular Subtyping of the Brain–Kidney Axis

Precision diagnosis extends beyond glomerular filtration rate (GFR)-based classification by incorporating transcriptomic and molecular profiling to define biologically distinct CKD phenotypes [152]. These subtypes, characterized by inflammation, fibrosis, or mitochondrial dysfunction, reflect differential activation of the brain–kidney axis. The kidney acts as a metabolic signaling organ, releasing uremic toxins such as IS and inflammatory mediators that disrupt BBB integrity and activate microglia. This bidirectional vulnerability is further supported by the strain vessel hypothesis, linking shared microvascular injury in renal and cerebral arterioles. Early biomarkers—including microalbuminuria and urinary cytokine signatures—serve as proxies of endothelial dysfunction and neuroinflammation [153,154], enabling detection of high-risk individuals prior to overt neurological or renal decline. Consistently, urinary peptidome profiling in CKD G3–G5 patients identified >1000 peptides that stratified individuals into distinct molecular clusters enriched for inflammatory and fibrotic pathways, while a 90-peptide signature accurately predicted 3-year kidney failure, supporting its value for precision risk stratification and mechanistic insight [155].

### 6.2. Multi-Omic Biomarkers

Integration of multi-omic biomarkers enables refined, mechanism-based risk stratification within the brain–kidney axis [156]. At the genomic level, whole-exome sequencing identifies monogenic drivers of early metabolic dysfunction and renal vulnerability [157]. This is complemented by transcriptomic profiling, which reveals compartment-specific gene expression signatures—including IFI16, COL3A1, ZFP36, NR4A3, DUSP1, FOSB, HBB, FN1, and PTPRC—across glomerular and tubular compartments in large integrative datasets [158].

At the metabolic level, metabolomic profiling captures gut–brain–kidney crosstalk, highlighting uremic toxins such as TMAO and IS, which activate AhR signaling and contribute to central autonomic dysregulation [159]. Beyond omics layers, functional biomarkers further reflect integrated pathophysiological states. Among early-life markers, ADMA plays a pivotal role by impairing nitric oxide bioavailability, reducing nephron endowment, and promoting oxidative stress [160,161]. Within the brain–kidney axis, elevated ADMA represents a shift toward a pro-oxidative “redox switch,” linking endothelial dysfunction to chronic sympathetic activation and neuroinflammation [56].

Similarly, urinary biomarkers associated with kidney injury and inflammation have been widely described [162,163]; however, their integration into a brain–kidney axis framework remains limited. Taken together, these findings support the development of composite multi-biomarker panels that capture bidirectional neuro–renal crosstalk and integrate complementary inflammatory, metabolic, and hemodynamic pathways to enhance predictive precision.

### 6.3. Digital Twins, AI-Driven Modeling, and Translational Challenges

These multi-omic and biomarker datasets provide the foundation for systems-level integration using artificial intelligence and digital twin technologies [164,165]. In current clinical practice, however, most applications remain limited to early-stage predictive analytics rather than fully realized digital twin systems. By incorporating clinical, omics, and imaging data, digital twins are under active development as simulation frameworks that aim to model how declining renal function and increasing uremic toxin burden may reshape cerebral connectivity, blood–brain barrier integrity, and sympathetic output.

AI-driven analytics further extend this framework by enabling probabilistic prediction of therapeutic response, thereby supporting phenotype-guided, mechanism-based treatment selection. At present, clinically implemented AI tools in CKM-relevant domains are largely restricted to risk prediction models (e.g., CKD progression [166], AKI risk [167], and cardiovascular event prediction [168]) rather than integrated multi-organ CKM simulators. In this context, emerging precision medicine models suggest that distinct CKM phenotypes may exhibit differential therapeutic responsiveness; for example, individuals with predominant inflammatory or neuroinflammatory signatures may preferentially benefit from GLP-1 receptor agonists [169], whereas those with dominant hemodynamic stress phenotypes appear more responsive to SGLT2 inhibitors, which primarily reduce intraglomerular pressure and modulate cardiorenal hemodynamics [170]. However, these phenotype–therapy matching strategies remain largely exploratory and have not yet been validated in prospective CKM-stratified clinical trials.

However, translation into routine clinical practice remains constrained by several key challenges. First, standardized and scalable biomarkers for clinical deployment are lacking; currently available markers (e.g., eGFR, albuminuria, NT-proBNP, HbA1c, hsCRP) provide only partial coverage of CKM biological complexity and lack sufficient specificity for digital twin calibration [171]. Second, the prolonged latency between early-life programming and overt disease complicates validation and implementation. Third, most redox and uremic toxin markers remain research-grade. Fourth, existing AI models are often trained on single-domain datasets (renal, cardiovascular, or metabolic) and lack cross-organ integration or external validation across diverse populations [172]. Finally, no single therapeutic strategy currently restores the integrity of the blood–brain barrier, glomerular filtration barrier, and gut–vascular barrier simultaneously.

Addressing these barriers will require integration of molecular subtyping, early-life biomarkers, and AI-driven modeling into clinically actionable decision frameworks, thereby bridging mechanistic insight with precision intervention. Future implementation will likely depend on stepwise clinical translation, beginning with validated single-domain risk models and progressively evolving toward integrated CKM digital phenotyping systems as multi-center longitudinal datasets become available [173].

## 7. Conclusions and Future Perspective

CKM syndrome should be conceptualized as a life-course disorder driven by persistent oxidative stress and redox imbalance within maladaptive brain–kidney crosstalk [174], necessitating a shift from single-target interventions toward integrated, systems-level strategies [2,3]. Rather than viewing cardiovascular, renal, and metabolic dysfunction as discrete entities, this framework emphasizes their dynamic interdependence, coordinated through redox-sensitive neurohumoral activation, immune signaling, and metabolic dysregulation. Multimodal approaches combining nutritional optimization, microbiome modulation, pharmacological therapies, and neurohumoral blockade offer synergistic potential to disrupt the oxidative-stress-driven feed-forward loops underlying multi-organ dysfunction. Such integrative strategies are particularly critical given the bidirectional amplification between central nervous system dysregulation and kidney injury, in which ROS act as key signaling mediators sustaining chronic disease progression across the lifespan. Importantly, while much of the mechanistic understanding is derived from experimental models, emerging human studies—including neuroimaging evidence of structural and functional brain alterations in CKD [175], clinical data on sympathetic overactivity and neuroinflammatory markers [176], and early-life cohort studies linking developmental exposures to later cardiorenal and neurocognitive outcomes [177,178]—provide convergent but still incomplete support for brain–kidney interactions in CKM syndrome.

Among these, precision nutrition and gut microbiota modulation represent central, early-intervention pillars of axis-targeted reprogramming, particularly during critical developmental windows [179]. Early-life exposures—including maternal nutrition, microbiota composition, environmental pollutants, and redox-disrupting factors—exert lasting effects on nephron endowment, BBB integrity, and neuroimmune development, thereby shaping long-term susceptibility to CKM syndrome. Dietary patterns such as the DASH and Mediterranean diets confer dual neuro–renal protection, in part by attenuating oxidative stress and reducing substrates for microbiota-derived uremic toxins. These diets also promote SCFA production, enhance antioxidant defenses, improve epithelial and endothelial barrier integrity, and modulate central inflammatory signaling, thereby reinforcing resilience across the brain–kidney axis. Furthermore, phenotype-guided dietary interventions may mitigate oxidative and mitochondrial perturbations associated with transient hyperglycemia and hepatic insulin resistance, highlighting the role of tailored nutritional strategies in modifying disease trajectories [180,181]. Collectively, these findings position precision nutrition not merely as supportive care, but as a redox-modulating therapeutic modality capable of reshaping disease programming at its earliest stages. However, evidence supporting developmental reprogramming interventions remains largely preclinical, and their efficacy, optimal timing, and long-term safety require validation in well-designed human studies.

In parallel, emerging precision medicine tools—including digital twin models and composite indices of brain–kidney connectivity—are poised to transform disease stratification and management [182,183,184]. By integrating longitudinal clinical data with multi-omic, imaging, and physiological inputs, these platforms enable real-time simulation of disease trajectories and individualized prediction of therapeutic response, including redox status and oxidative stress burden. Complementary experimental platforms, such as in vitro multi-organ-on-a-chip systems, further enable mechanistic dissection of inter-organ crosstalk, particularly along the brain–kidney axis [185]. These systems provide controlled environments to interrogate causal pathways, including ROS signaling, mitochondrial dysfunction, and antioxidant response networks, validate candidate biomarkers, and test targeted interventions across interconnected organ systems. Integration of multimodal data, including functional neuroimaging and renal dynamic assessments, allows quantitative characterization of axis-level dysfunction and early detection of oxidative-stress-dominant subphenotypes, facilitating deployment of personalized, mechanism-based combination therapies. Importantly, this convergence of computational and experimental platforms establishes a translational bridge between systems biology insights and clinical decision-making.

Looking forward, reprogramming strategies for CKM syndrome define a proactive, life-course-oriented paradigm centered on early intervention and risk modification. This paradigm shifts the clinical focus from late-stage disease management to upstream prevention, targeting critical windows of vulnerability during fetal development, infancy, and early childhood. By targeting shared neurohumoral, metabolic, inflammatory, and redox-regulated pathways, these approaches aim to prevent the establishment and propagation of maladaptive programming. Interventions that restore redox homeostasis, enhance endogenous antioxidant capacity, preserve barrier integrity, and normalize neuroimmune signaling may exert cascading benefits across multiple organ systems, thereby attenuating long-term disease burden. Future translational and clinical studies are needed to determine whether the mechanistic insights and therapeutic benefits observed in animal models can be replicated in human populations across the life course.

The coordinated integration of nutritional, microbial, and pharmacological interventions—applied with temporal and mechanistic precision—holds promise for interrupting intergenerational transmission of risk and achieving sustained improvements in cardiovascular–kidney–metabolic health. Ultimately, the convergence of precision nutrition, systems biology, and AI-driven modeling may facilitate a paradigm shift toward predictive, preventive, and personalized medicine. By integrating redox biology with interconnected neurohumoral, inflammatory, metabolic, and organ-crosstalk pathways, these approaches may improve risk stratification and therapeutic precision, thereby transforming CKM syndrome from a progressive multisystem disorder into a more modifiable and potentially reversible condition across the life course.

## Data Availability

No new data were created or analyzed in this study.

## Abbreviations

The following abbreviations are used in this manuscript:

ADMA	Asymmetric dimethylarginine
AhR	Aryl hydrocarbon receptor
BBB	Blood–brain barrier
CBRNA	Catheter-based renal nerve ablation
CKMS	Cardiovascular–kidney–metabolic syndrome
CKD	Chronic kidney disease
CVD	Cardiovascular disease
DOHaD	Developmental Origins of Health and Disease
GLP-1Ras	GLP-1 receptor agonists
HPA axis	Hypothalamic–pituitary–adrenal axis
IS	Indoxyl sulfate
MASLD	Metabolic dysfunction-associated steatotic liver disease
pCS	p-cresyl sulfate
POMC	Pro-opiomelanocortin
RAS	Renin-angiotensin system
SCFA	Short-chain fatty acid
SGLT2	Sodium–glucose cotransporter-2
TMAO	Trimethylamine N-oxide
